# Mechanisms underlining R-loop biology and implications for human disease

**DOI:** 10.3389/fcell.2025.1537731

**Published:** 2025-02-21

**Authors:** Junzhe Liu, Fengze Li, Yulong Cao, Yonghui Lv, Kunjian Lei, Zewei Tu, Chuandong Gong, Haiyan Wang, Feng Liu, Kai Huang

**Affiliations:** ^1^ Department of Neurosurgery, The 2nd Affiliated Hospital, Jiangxi Medical College, Nanchang University, Nanchang, Jiangxi, China; ^2^ Institute of Neuroscience, Nanchang University, Nanchang, Jiangxi, China; ^3^ College of Queen Mary, Nanchang University, Nanchang, Jiangxi, China; ^4^ Department of Operation, The 2nd Affiliated Hospital, Jiangxi Medical College, Nanchang University, Nanchang, Jiangxi, China; ^5^ Department of Neurosurgery, Jiangxi Children’s Hospital, Nanchang, China

**Keywords:** R-loop, replication-transcription collision, genomic instability, neurodegenerative disorders, gene regulation, cancer

## Abstract

R-loops are three-stranded non-canonical nucleic acid structures composed of nascent RNA hybridized with the template DNA strand, leaving the non-template DNA strand displaced. These structures play crucial roles in regulating gene expression, DNA replication, and transcription processes. However, R-loops have also been increasingly described as highly deleterious, causing genomic instability and DNA damage. To maintain R-loops at a relatively safe level, complex regulatory mechanisms exist to prevent their excessive formation. The growing understanding of R-loop functions has provided valuable insights into their structure and potential clinical applications. Emerging research indicates that R-loops contribute to the pathogenesis of various disorders, including neurodegenerative, immune-related, and neoplastic diseases. This review summarizes R-loop metabolism and its significance in the etiology of associated disorders. By elucidating the regulatory mechanisms governing R-loops, we aim to establish a theoretical foundation for understanding disease pathogenesis and exploring novel therapeutic strategies targeting these hybrid nucleic acid structures.

## 1 Introduction

**FIGURE 1:**
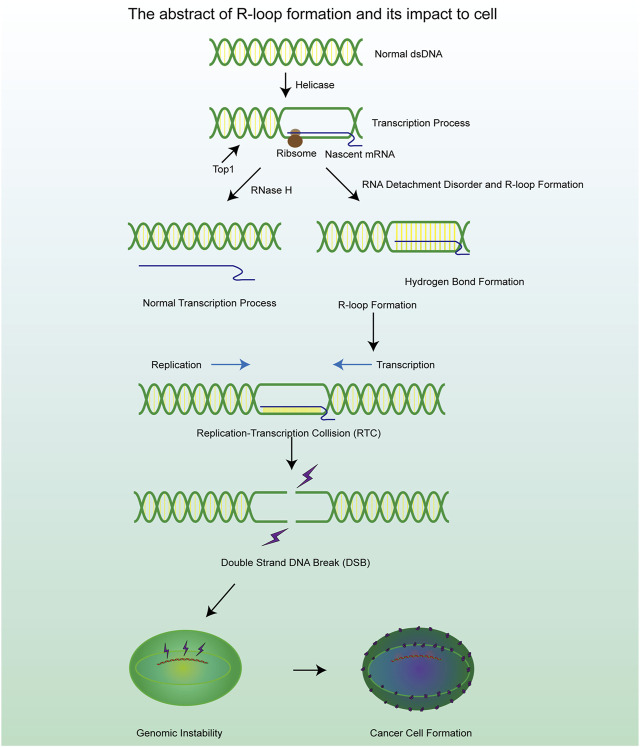
A brief mechanism diagram of the formation process of R-loop and DSB process, which contribute to the cell genomic instability and cancer cell formation.

**FIGURE 2:**
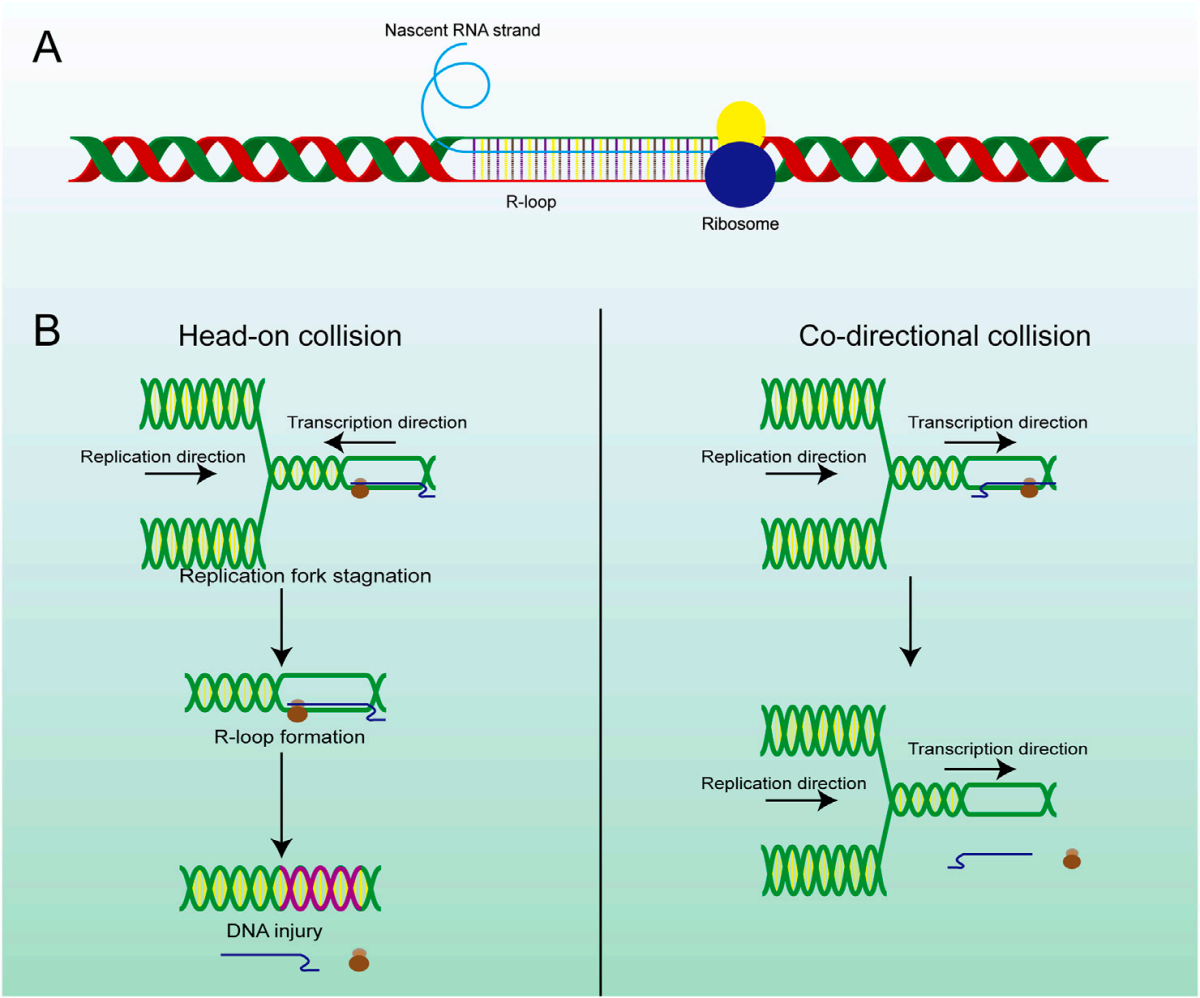
The structure and main formation mechanism of R-loop. **(A)** Display diagram of simulation structure of R-loop: nascent RNA strand invades DNA double-strand to form R-loop. **(B)** The difference between head-on RTC and co-directional RTC and the different consequences, head-on collision is more dangerous than co-directional collision and is highly related to R-loop and subsequent gene damage.

R-loops are structures frequently generated during DNA replication, DNA repair, and especially transcription. These three-stranded nucleic acid structures consist of RNA-DNA hybrids and a displaced single-stranded DNA. Alternatively, R-loops can be viewed as nascent RNA invading double-stranded DNA, forming a more stable three-stranded nucleic acid structure (Figure 2A) (Chedin and Benham, 2020; Maffia et al., 2020). R-loops are widely observed in the genomes of both prokaryotic and eukaryotic cells, including bacteria, yeast, and human cells (Drolet and Brochu, 2019). The R-loop structure is highly stable under physiological conditions, maintaining hybridization even after restriction endonuclease (RE) treatment, rendering it non-degradable and persistent within the cell. Thermodynamic analysis revealed the structure of R-loops was more stable than that of double-stranded DNA (dsDNA), which makes it difficult for the formed R-loops to automatically recover to dsDNA and this is a requirement for the formation of R-loops. Since R-loops are extremely stable and hard to degrade in cells, many factors are required to remove redundant and harmful R-loops. Recent studies have revealed numerous positive functions of R-loops, which are extensively involved in key biological processes such as transcriptional activity maintenance, genome replication, and chromosome structure remodeling (Castellano-Pozo et al., 2013; Ginno et al., 2012). This has led to the realization that R-loops are indispensable in cells and make a significant contribution to the maintenance of normal cellular physiological activities. Paradoxically, although R-loops do play essential roles in many cellular processes, they are also important hazard factors for DNA damage and genomic instability which can result in seriously harmful consequences in the cells (Figure 1) (Stork et al., 2016). The latest studies suggest that the excessive accumulation of R-loops is closely related to the occurrence of various diseases including neurodegenerative diseases, immune-related diseases and even cancers (Crossley et al., 2023; Elsakrmy and Cui, 2023; Xu et al., 2023). Therefore, the excessive accumulation of R-loops must be tightly controlled to maintain genomic stability, which may receive unexpected benefits for the treatment of related diseases.

Although the concept of R-loops has been proposed for several decades (Drolet et al., 1994), related research about R-loops is still in its relative infancy. With the advances in experimental and detection methods, the structure and functions of R-loops have been revealed gradually. We found that R-loops occur not only in the transcription initiation and termination regions but also in other cellular processes. The formation of regulatory R-loops in certain regions could enhance some physiological functions (Niehrs and Luke, 2020). For example, in the immune system, R-loops can promote class switch recombination (CSR) in vertebrates, which may allow plasma cells to produce antibodies of different types and functions to fight various pathogens and maintain the stability of the cellular immune system (Yu and Lieber, 2019). In addition, R-loops also promote the process of mitochondrial DNA replication, CRISPR-Cas9 gene editing, specific regulatory steps in transcription initiation and termination, telomere homeostasis and bacterial plasmid replication. The functions mentioned above are sufficient to prove the status of R-loops as cell regulatory factors and will be described in detail below. However, unlike DNA or RNA, R-loops exert harmful influences on cells. In certain circumstances, such as the loss of enzymes and functional proteins, it is more likely to cause the excessive accumulation of intracellular R-loops and have significant damage to the cell status. For example, numerous R-loops are formed at the transcription factor binding regions, which obstruct the transcription factor binding sites and consequently inhibit the transcription process (Li et al., 2020). Generally speaking, the presence of R-loops in cells is transient. This is because cells possess various mechanisms for resolving R-loops, which help maintain a dynamic equilibrium in the number of R-loops by precisely regulating their degradation process (Cerritelli and Crouch, 2009).

The methods of R-loop detection have also developed rapidly in recent years. The most typical method to detect the existence of R-loops is the utilization of S9.6 antibody. A monoclonal antibody (S9.6) binds with R-loops specifically and is highly sensitive to the detection of RNA-DNA hybrids with 0.6 nm affinity which makes it possible to detect R-loops both in cells and *in vitro* (Hartono et al., 2018; Gibbons and Aune, 2020). DNA-RNA immunoprecipitation (DRIP) and immunofluorescence methods are based on the robust binding between S9.6 and DNA-RNA hybrids and they are the predominant technology used to evaluate the level of R-loops in cells (Garcia-Rubio et al., 2018). However, S9.6 not only exhibits a high affinity for DNA-RNA structures but also has a very close binding with double-strand RNA. This means that S9.6 lacks specificity in the detection of R-loops and usually requires the establishment of a control group for detection, which greatly increases the workload of detection and leaves questions about accuracy (Phillips et al., 2013). To address this issue, some studies have improved the reliability and specificity of antibody-based detection by incorporating multiple control steps. These involved the use of RNase H, RNase T1 and RNase III to cleave DNA-RNA hybrids, single-stranded RNA and double-stranded RNA (Ramirez et al., 2021). This multi-pronged approach helps to enhance the confidence level of the antibody-based assays. After the detection of R-loops in cells, the template DNA sequence of the R-loops can be further sequenced by high-throughput sequencing (DRIP-seq) and the cDNA strand obtained by reverse transcription of the separated RNA strand can be sequenced (DRIPc-seq) to understand the sequence characteristics of the R-loops (Sanz and Chedin, 2019; Malig and Chedin, 2020; Chen et al., 2017a), high-resolution DRIP-seq shows that R-loop is indeed a widespread and abundant structure in the genome, which may accounting for about 5% of the human genome and 8% of the yeast genome (Wahba et al., 2016). Although the above methods can be used for preliminary determination of R-loops content and location in cells, these techniques are not simple and precise enough and therefore they have not been widely used and their clinical application value is very limited. However, Chen, Liang et al. confirmed the critical biochemical properties of the R-loop using RNase-H by expressing the catalytically inactive RNASEH1 and performing strand-specific amplification of immunoprecipitated (IPed) DNA (termed R-ChIP), which effectively captured the relevant R-loop (Chen et al., 2017b). Not coincidentally, catalytically inactive, purified RNase H1 has also been shown to be reliable in the detection of DNA-RNA hybrids, and is expected to become a universal detection tool (Crossley et al., 2021). The discovery of these novel R-loop assays provides important technical support for future progress in R-loop-related research.

## 2 Metabolism of R-loop in cells

R-loops are normal intracellular products in cells, and most R-loops are generated during the process of transcription. The occurrence of R-loops is related to various factors, including the GC content in the DNA sequence, DNA topology, and the abundance of related enzymes (Belotserkovskii and Hanawalt, 2022). During transcription, the GC-rich DNA strand is transcribed by RNA polymerase II, producing a GC-rich RNA. The three hydrogen bond connections between C and G bases enable the stable formation of the DNA-RNA hybrid structure, making it resistant to dissociation (Petermann et al., 2022). Many enzymes play crucial roles in DNA-associated physiological activities, and the absence of relevant helicases or RNases is a major source of R-loop formation (Yang et al., 2023; Templeton and Laimins, 2023). Furthermore, specific nucleic acid structures in the genome also contribute to the formation of R-loops. The process of R-loop formation is not the consequence of a single factor but rather the product of the combined action of multiple factors. The main cause that promotes the formation of the R-loop is as follows.

### 2.1 DNA sequence

The DNA sequence can exert a limited influence on the formation of R-loops. Recent scientific research has demonstrated that the R-loop is easier to form on DNA sequences with high GC content, especially when GC-rich DNA sequences are transcribed to produce GC-rich transcripts (Ginno et al., 2013). Generally speaking, the distribution of various deoxynucleotides varies greatly due to the universality of genetic information on the DNA strand. G-rich DNA fragments, also called G clusters, are proved to be robust initiation sites for the formation of R-loop (Shukla et al., 2022). Apart from that, the RNA transcribed by Pol II from the telomere terminal region usually contains -UUAGGG-repeats and they are called telomeric repeat-containing RNA (TERRA). The sequence characteristics of TERRA could be regarded that a nascent RNA invades dsDNA to form the R-loop (Feretzaki et al., 2020). The formation of R-loops at telomeres enhances telomere stability, but the presence of R-loops also inhibits telomere replication, potentially triggering genomic instability (Bettin et al., 2019). As an unmethylated promoter sequence often found in the human genome, CpG islands (CGI: C-G and G-C rich sequences) manifests a significant strand asymmetry in the distribution of guanines and cytosines, a property known as GC skew. Such a property also makes it a hotspot for R-loop formation (Ginno et al., 2012).

### 2.2 DNA secondary structure

**FIGURE 3:**
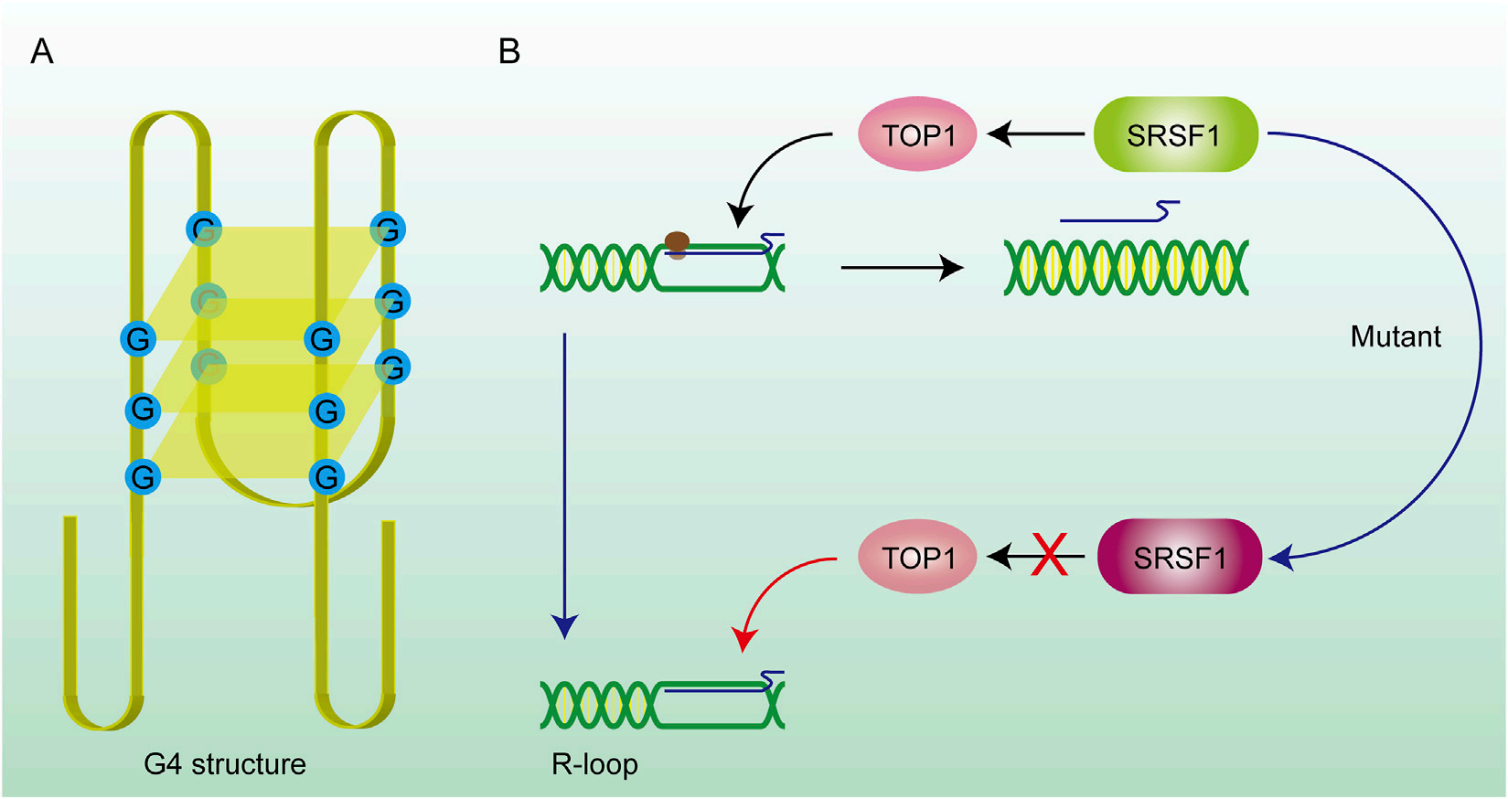
DNA secondary structure and gene mutations promote R-loop formation. **(A)** G4 structure: The G4 structure consists of hydrogen bonds formed between four guanines on the same DNA strand, forming a quadrilateral plane, and potassium ions can stabilize the structure on the plane. The existence of this structure prevents the DNA strand from being stretched and hinders the DNA replication and transcription process. RNA is stuck on the DNA strand to form an R-loop. **(B)** SRSF1 recruits TOP1 on the R-loop and promotes RNA to drop off from the template chain. After the SRSF1 mutation, this effect is lost and the R-loop abnormally accumulates.

Some DNA secondary structures are also involved in the stabilization of R-loops. G-quadruplex (G4) structures (Figure 3A) are composed of four guanine bases through the Hoogsteen base pairing principle and are mainly formed on the single strand DNA (ssDNA) (Wanrooij et al., 2012; Lee et al., 2020). Scientific research shows that the loss of ATRX contributes to the formation of G4 structures and R-loops at telomeres (Nguyen DT. et al., 2017). Stabilization of the G4 structure is enhanced by the binding of G4 ligands which renders R-loops more persistent in the genome. In addition to playing negative roles in the genome through the R-loops pathway, the G4 structures also inhibit the DNA replication and transcription process directly which causes genomic instability (Chambers et al., 2015; Tan et al., 2020a). The G4 structure is rooted in guanine nucleotides and is limited by the structure of chromatin and a monovalent cation (M^+^) on the guanylate plane contributes to the stability of the G4 structure (Miglietta et al., 2020; Cadoni et al., 2021). Reactive oxygen species (ROS) could also promote the production of R-loops through inducing the production of G4 structures in cells and could be further enhanced through G4 ligands (Wu et al., 2023).

### 2.3 Transcription-replication collision (TRC)

TRCs are a critical pathological process closely associated with genome stability and cell viability, which is another potential pathway for excessive R-loop accumulation (Stoy et al., 2023). As we know, replication and transcription can occur simultaneously in the same genomic regions, and inappropriate regulatory mechanisms may result in TRCs, which can stall both processes and lead to further genome damage (Hamperl et al., 2017). The observed aberrant gene expression phenomenon occurs in both eukaryotic and prokaryotic cells (Browning and Merrikh, 2024). Depending on the directionality of the two processes, TRCs can be categorized into two types co-directional collisions and head-on collisions (Figure 2B). In head-on collisions, where replication and transcription proceed in opposite directions, the detrimental effects are more pronounced, and the accumulation of R-loops is likely to form under these conditions (Belotserkovskii et al., 2018). Co-directional collision occurs when transcription is in the same direction as the replication fork, the transcriptome follows the replication fork and the number of R-loops produced is greatly reduced (Hamperl et al., 2017). Consequently, damage to the genome will also be markedly reduced. This process is the result of the interaction and co-regulation of multiple genes (Lin and Pasero, 2017). Inhibiting BRD4 expression can increase the formation of R-loops and facilitate TRC, DNA damage, and cell death mediated by replication stress and fork slowing. By controlling the expression of specific genes, the possibility of TRC occurrence is artificially reduced, thus regulating the production and accumulation of R-loops (Lam et al., 2020a; Zhang et al., 2018).

### 2.4 Splicing factor and relevant RNase

SRSF1 (Serine/Arginine-rich Splicing Factor 1) is a typical splicing regulatory factor that can excise longer pre-mRNA transcripts, generating shorter mature mRNA molecules (Li et al., 2023). This process accelerates the dissociation of mRNA from the DNA template, thereby facilitating the renaturation of the double-stranded DNA. However, some of the longer pre-mRNA transcripts fail to be properly spliced into multiple shorter mature mRNA molecules when SRSF1 is mutated. These unspliced pre-mRNA transcripts often contain enriched G-cluster sequences, which prevent the mRNA from dissociating from the template strand, leading to the formation of R-loop structures (Petermann et al., 2022). A mutant SRSF1 also affected the recruitment of Top I, which made the dsDNA highly twisted and eventually led to double-strand breaks (DSBs) (Figure 3B) (Girstun et al., 2019), suggesting that targeting aberrant SRSF activity to correct faulty RNA and DNA metabolism can have significant therapeutic benefit. Mutations in splicing factors (e.g., SF3B1) can promote dsDNA breakage and neuronal cell apoptosis followed by constant accumulation of R-loops (Sorrells et al., 2018). In summary, mutations in numerous splicing-related genes will facilitate the formation of R-loops. Therefore, splicing abnormalities are closely related to genome instability and affect various cellular processes (Nguyen et al., 2018).

### 2.5 Binding protein of RNA and ssDNA

Some binding proteins are also proven to be potential facilitators in the formation of R-loops. Changes in relative protein content can also affect the synthesis and accumulation of R-loops. After the formation of ssDNA in eukaryotic cells, some proteins will be present that maintain the DNA in the single-stranded state, such as replication protein A (RPA), which is very common in eukaryotic cells and considered the protective protein of ssDNA. RPA was proved to coordinate DNA replication by assisting in the recruitment of Timeless-Tipin (Tim-Tipin) complexes to replication forks, forming Tim-Tipin-RPA-ssDNA complexes (Witosch et al., 2014). In the process of DNA transcription, RPA has a strong binding affinity with both DNA and RNA. Furthermore, RPA cooperates with and enhances the activity of RNase H1, an enzyme that binds closely with DNA-RNA hybrids and removes the RNA strand in the DNA-RNA hybrid (Nguyen HD. et al., 2017). As an RNA-binding protein, TDP-43 contains highly conserved RNA recognition motifs (RRMs) that tightly bind to RNA strands (Kuo et al., 2014). The TDP-43 (TAR DNA-binding protein) can promote the shedding of RNA from the template strand and prevent its cross-linking with ssDNA due to its ability to bind RNA. The accumulation of R-loops due to the absence of TDP-43 induced DNA damage and compromised cell viability, which are considered significant hallmarks of neurodegenerative diseases (Prasad et al., 2019). Beyond that, there are many other proteins involved in the regulation of R-loop formation. Since the mechanism of action is similar, we will not go into details here.

### 2.6 R-loops in mitochondria

DNA is present not only in the nuclear genome of cells, but also within the mitochondrial organelles. This allows mtDNA (mitochondrial DNA) to self-replicate as cells divide and proliferate, laying the foundation for the circular structure of mtDNA. In fact, studies have detected the expected R-loop in the control region of mtDNA (Akman et al., 2016). The R-loop is primarily formed during the initiation of mtDNA replication, utilizing short RNA molecules as primers for the lagging strand extension. RNaseH1 is a key player in mitochondrial R-loop metabolism since it degrades RNA only upon hybridization to DNA and can remove R-loop structures, suggesting that the absence of the mitochondrial RNaseH1 may enables the formation of mitochondrial R-loops (Holt, 2019). Interestingly, due to its inclusion of an DNA-RNA heterodimeric binding structural domain, RNase H1 may also exhibit a protective effect on the mitochondrial R-loop (Uhler et al., 2016). The R-loop is crucial for the mtDNA replication process, but an excessive accumulation of R-loops can lead to instability of the mitochondrial genome. The ability of the mitochondria-specific helicase SUV and the ribonucleotide nucleotidyltransferase 1 (PNPT1) to degrade mitochondrial RNA may have a positive effect on the balance of mitochondrial R-loop accumulation (Shimada et al., 2018; Wang et al., 2009).

### 2.7 Enzymes correlate with R-loops formation

Maintaining a balanced level of R-loops in the cell is essential, and one of the simplest measures to achieve this goal is to maintain the activity of the enzymes involved in the biochemical processes. Multiple enzymes are involved in the metabolism of R-loops, and they are the most important factors in regulating R-loop degradation and preventing its accumulation. These enzymes include DNA-RNA helicases, RNase H, and topoisomerases, and their stability is critical because fluctuations in their levels directly affect the steady-state concentration of R-loops in the cell. For example, DNA-RNA helicase is responsible for unwinding the DNA-RNA hybrids that form the R-loop, thereby facilitating the breakdown of the R-loop. On the other hand, RNase H catalyzes the hydrolysis of RNA molecules within the R-loop, leading to its disassembly. Meanwhile, topoisomerases play an important role in relieving the torsional stress associated with the R-loop structure and preventing its further propagation. Disturbances in the regulation of these enzymes can lead to dysregulation of R-loop homeostasis, which may contribute to a variety of pathologies. Therefore, maintaining the stability and normal function of these key enzymes is a critical measure to ensure the proper regulation of R-loop homeostasis in the cellular environment. This is described in the following section.

#### 2.7.1 RNaseH

RNaseH is considered the primary enzyme responsible for the direct degradation of various RNAs within cells and is also essential for maintaining cellular genome stability. There are two types of RNaseH in cells: RNase H1 and RNase H2. Both are capable of resolving the RNA strand in DNA-RNA hybrid structures by removing the RNA primers from Okazaki fragments, while RNase H2 also participates in ribonucleotide excision repair (RER) (Hyjek et al., 2019). Modulating the expression of cellular RNaseH to regulate the levels of R-loops has become a well-recognized fact (Camino et al., 2023). Reduced R-loop levels were observed through the detection of the S9.6 antibody in the knockdown assay of RNaseH (Drolet et al., 1995). The absence of RNaseH means that cells lose the ability to modify RNA, and the strong hydrogen bonds between nascent RNA and template DNA would tightly bind the two strands, which is a major contributor to R-loop formation. In fact, RNase H is a key enzyme that degrades the RNA moiety of R-loops, thereby preventing the accumulation of R-loops (Camino et al., 2023). Studies have shown that R-loop levels in RNase H1 knockout mice are elevated, leading to impaired mitochondrial function and liver degeneration (Lima et al., 2016). Furthermore, in cells where RNase H1 activity is absent, R-loop levels increase, resulting in DNA damage and replication fork stalling (Zhang W. et al., 2024). These findings highlight the crucial role of RNase H in maintaining genomic stability by resolving R-loops.

#### 2.7.2 ATP-dependent helicases

**FIGURE 4:**
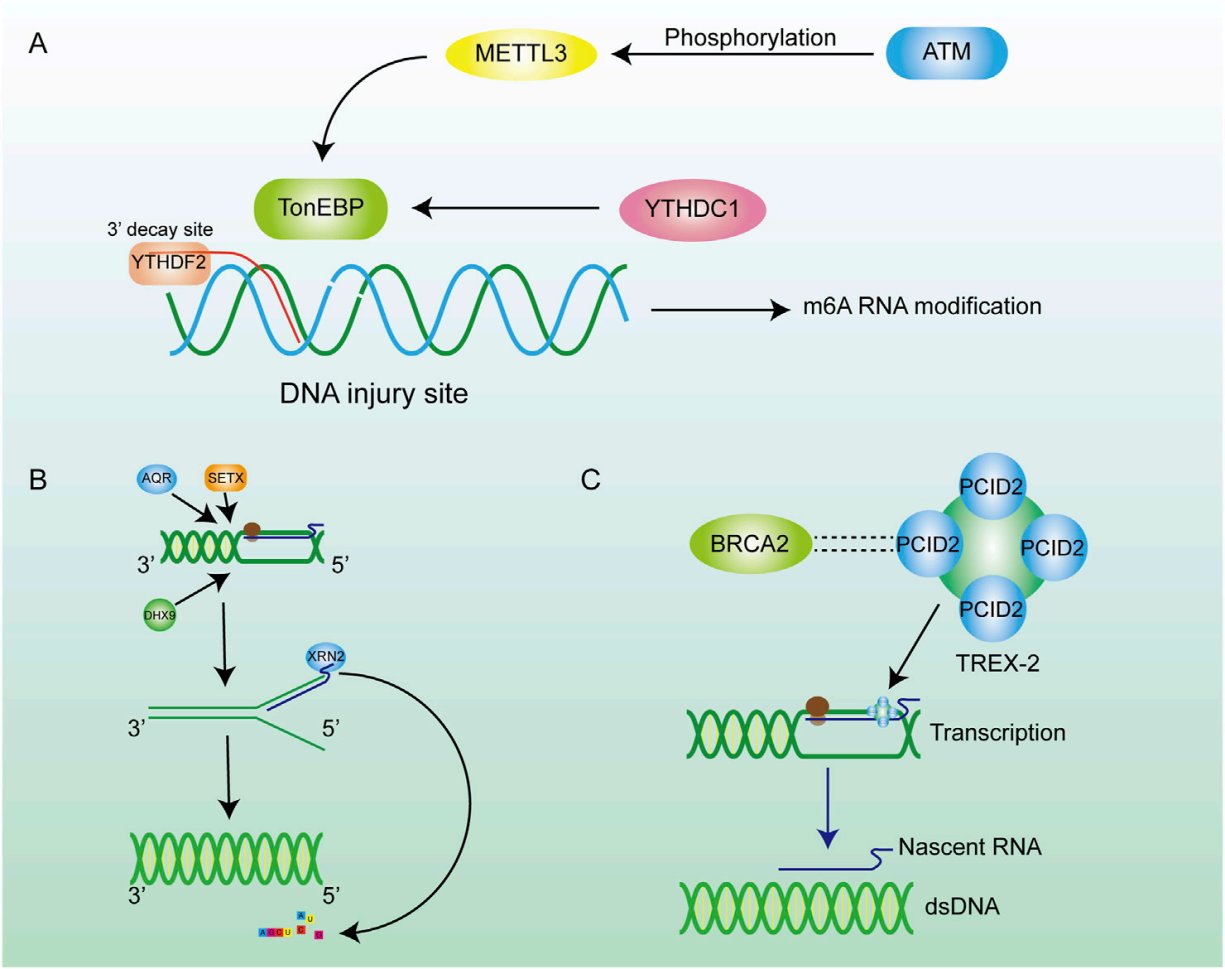
Inhibition of R-loop formation by m6A pathways and enzymes. **(A)** ATM phosphorylation activates METTL3 to regulate m6A methylation through the METTL3-m6A-YTHDC1 axis, thereby inhibiting further damage of hybrid nucleic acids. **(B)** Many enzymes are involved in the regulation of R-loop content in cells. Helicase removes the DNA double helix, promotes the transcription process, and encodes XRN2 on RNA to promote RNA degradation. **(C)** The BRCA family can maintain chromosome stability by combining with other proteins.

Although the mechanism of degrading R-loops in cells by RNaseH can achieve satisfactory results, it is a significant waste to hydrolyze the nascent RNA chain, especially the long-chain RNA. Many reports have revealed that numerous RNA-dependent ATPases are involved in the unwinding of RNA-DNA hybrid duplexes (He et al., 2022). This class of ATP-dependent helicases includes senataxin (SETX) (Suraweera et al., 2007), Aquarius (AQR) (Sollier et al., 2014), and DEAD-box helicase (DDX) family members such as DDX5, DDX41 (Shinriki and Matsui, 2022; Mersaoui et al., 2019). They are the second largest class of enzymes that remove R-loops after RNase H. SETX primarily removes the R-loop generated in the transcription termination region. AQR possesses 3′-5′ RNA helicase activity and single-stranded RNA binding activity, which can release the RNA strand through untwisting and facilitate mRNA splicing. DDX19 enables RNA binding activity and RNA helicase activity, and it participates in the mRNA export from the nucleus (Arul Nambi Rajan and Montpetit, 2021). In addition to above helicases, some novel DNA helicases have been continuously identified, such as DHX9, which has been found to be a driver for R-loop formation, as it releases the nascent RNA to produce a dissociative RNA terminal which can then combine with single-stranded DNA during the transcription process, leading to the formation of R-loops (Cristini et al., 2018). The phosphorylation of the DHX9 gene by ATR was required for the formation of R-loops (Liu et al., 2024). Evidence above indicates that DHX9 is an effective inducer of R-loops formation. In addition, DNA-RNA helicase AQR, SETX, and DHX9 could synergistically degrade the R-loops at the 3′-ends of a gene sequence. These three enzymes are important in transcriptional termination and the DNA damage resulting from the unstable abundance of R-loops (Figure 4B) (Cristini et al., 2018; Sakasai et al., 2017). The above three enzymes induce the untwisting of RNA-DNA hybrids, and the detached RNA strand is subsequently degraded by exonucleases such as XRN2. (Morales et al., 2016). The decrease in RNA abundance within the nucleus reduces the formation of DNA-RNA hybrid strands. Helicases degrade R-loops while retaining the original RNA, which is more conducive to the rational distribution of energy. These helicases are involved in the regulation of R-loops metabolism, and changes in their expression would also alter the abundance of R-loops.

#### 2.7.3 DNA topoisomerases

In addition to acting directly on R-loops to affect their metabolism, cells also prevent production of R-loop by modulating chromatin structure. DNA topoisomerases regulate and alter the topological states of chromatin by catalyzing the transient breaking and reconnection of DNA strands, thereby ensuring the proper release of supercoiling and torsional tension on the DNA. Recent research on the HeLa cell has suggested that the knockdown of TopI increased the abundance of R-loops by inhibiting the supercoiling that occurs during the transcription process (Promonet et al., 2020). This is consistent with the previously described fact that the excessively supercoiled structure contributes to the formation of R-loops. The enzymes mentioned have different physiological characteristics, but they prevent the damage caused by R-loops by inhibiting their synthesis and promoting their degradation. Apart from working independently, these enzymes cooperate to maintain the R-loop content. For example, a sustained lack of TopI leads to replication stress and replication-transcription collision (RTC), but this could be eliminated by overexpression of RNase H (Manzo et al., 2018; Lang et al., 2017).

### 2.8 RNA modification

Many kinds of RNA modifications contribute to the regulation of gene translation and the protein stability. As a common RNA modification, m6A methylation plays a crucial role in post-transcriptional modification (PTM) (He et al., 2019; Wang T. et al., 2020), which contributes to the metabolism of mRNA (Figure 4A). As an important m6A reader, YTHDF2 can recognize the site where m6A occurs on RNA (Wang et al., 2014). Similarly, the RNA strand in the R-loop can also be specifically recognized by YTHDF2. M6A methylation occurs on the RNA strand in the R-loop, and then the RNA strand bound to the template DNA strand is degraded through the m6A methylation mechanism (Kang et al., 2021). This process can loosen the DNA-RNA hybrid, reconnect the separated DNA strand to form dsDNA so that R-loop can be degraded. Scientists observed the increased R-loop levels, cell-growth retardation and cell morphology changes in the YTHDF2 knockdown assay (Abakir et al., 2020), which enhance the conclusion that m6A contributes to the degradation of the R-loop in cells. In addition to the functions mentioned above, m6A can partly control genome stability through the METTL3-m6A-YTHDC1 axis (Zhang J. et al., 2024). RNA methyltransferase METTL3 is activated by ATM phosphorylation when DSBs occur in cells. Activated METTL3 precisely locates the site of the DSBs and m6A methylation occurs at the site, which subsequently leads to recruitment of the m6A reader protein YTHDC1 to protect the affected site from further damage. The METTL3-m6A-YTHDC1 regulatory axis prevents the accumulation of DNA-RNA hybrid in cells and avoids further damage to the genome (Zhang et al., 2020). m5C also acts as an important PTM site, and its modification responds positively to DSB repair processes and extensively influences the regulation of R-loops metabolism (Chen et al., 2020). Chen et al. reported that the RNA methyltransferase TRDMT1 was recruited to DNA damage sites, facilitating the induction of m5C, which promoted the recruitment of DNA repair factors as an unexpected DNA damage response mechanism to regulate DNA repair. Furthermore, the m5C modification exhibited varying degrees of DNA-RNA hybrid affinity, revealing the latent regulatory potential of m5C for R-loop formation (Chen et al., 2020).

### 2.9 BRCA gene family

As tumor suppressor genes, BRCA gene families are essential for maintaining chromosomal stability, indicating their potential to protect the genome from damage (Bhatia et al., 2014; Dias Nunes et al., 2023). For example, BRCA1 is crucial for preserving telomere integrity, as it physically interacts with TERRA RNA in an R-loop-dependent manner, thereby inhibiting the formation of R-loops at telomeres (Vohhodina et al., 2021). Meanwhile, BRCA1 interacts with the protein SETX to form a complex recruited to R-loop-enriched termination regions of actively transcribed genes. The BRCA1/SETX complex mitigates co-transcriptional DNA damage resulting from unresolved R-loops at these loci (Hatchi et al., 2015). BRCA2 interacts with RNA polymerase II (RNAPII) to regulate its promoter-proximal pausing (PPP), preventing the formation of unexpected DNA-RNA hybrids (Shivji et al., 2018a). Likewise, BRCA2 has a close relationship with the TREX-2 component PCID2, and TREX-2 promotes the exportation of mRNA to prevent it from cross-linking with ssDNA in the nucleus to produce R-loops (Figure 4C) (Gondo et al., 2021). In conclusion, the BRCA families inhibite the formation of R-loops during telomere synthesis, DNA transcription, and other biological processes, thereby mitigating their detrimental effects on the organism.

## 3 The functions of R-loops in cells

### 3.1 Beneficial roles

R-loops were initially considered to be a non-functional transcriptional by-product with no substantial physiological function, but this conclusion was quickly overturned. Evidence for R-loops involvement in gene regulation was presented and researchers realised that R-loops could be a novel tool in gene regulation (Aguilera and García-Muse, 2012). The latest research has continued to reveal the regulatory function of the R-loop, which has deepened our understanding of the R-loops and contributed to new insights into the genetic landscape.

Judging from the existence form of R-loops in cells and their influence on the cell genome, R-loops were divided into physiological R-loops and pathological R-loops artificially (García-Muse and AguileraLoops, 2019). Physiological R-loops are related to a wide range of cellular processes, but pathological R-loops are one of the main sources of DNA damage and DNA genomic instability (Niehrs and Luke, 2020; Huertas and Aguilera, 2003). The formation of physiological R-loops usually relies on a programmed process, in which specific factors are required to ensure their formation (Belotserkovskii et al., 2018). However, their existence as R-loops in cells is transient as various enzymes like RNaseH 1/2 could dissociate the DNA-RNA hybrid (Cerritelli and Crouch, 2009; Amon and Koshland, 2016). Therefore, physiological R-loops are also widely regarded as intermediates of certain cellular processes. Pathological R-loops are mainly secondary to the loss of related gene expression, resulting in the persistent existence of R-loops at specific loci and degradation barriers of physiological R-loops. The formation of pathological R-loops is divorced from normal cellular functions and is not regulated by the genome. The persistence of pathological R-loops often causes severe damage to the genome, such as DNA replication and transcription process blockage. The DNA damage and genome instability further increase the probability of various diseases or even cancer.

#### 3.1.1 Immune boost

The function of R-loops varies greatly in different cell states, cell stages, and cell types. With the deepening of R-loops research, multiple functions of R-loops have been explored and fully elaborated. One typical example is the function of R-loops in class-switch recombination (CSR), which contributes to the diversity of antibodies. At the beginning of CSR in plasma cells, the R-loop is formed in the G-rich region of the IgH locus during the transcription process of antibody synthesis (Wiedemann et al., 2016). The DNA-RNA hybrid can free a long and stable ssDNA, enhancing the binding force of activation-induced deaminase (AID) to ssDNA (Yu and Lieber, 2003). The enzyme can deaminate cytosine residues on ssDNA to form uracil, which is then processed by base excision repair factors into DSB (Harris et al., 2002). After being repaired by non-homologous end-joining (NHEJ), intrachromosomal deletional recombination can occur to produce different kinds of immune proteins and improve immunity (Yu and Lieber, 2019; Stavnezer et al., 2008). In addition, there are indications that the R-loop is also implicated in immune signaling (Brickner et al., 2022). Because intracellular recognition of non-self and self nucleic acids can lead to the initiation of potent pro-inflammatory and anti-viral cytokine responses, the R-loops could be specifically recognized by cGAS and TLR9 thereby facilitating immune signaling, which has recently been found to promote the innate immune response in humans by activating IRF3 (Crossley et al., 2023; Mankan et al., 2014; Rigby et al., 2014).

#### 3.1.2 DNA supercoils eliminator

The role of R-loops in DNA topology has also been gradually revealed (Mackay et al., 2020). As we know, in the process of DNA transcription and replication, in order to promote the efficiency of dsDNA unwinding, a negative superhelix is formed on the DNA strand and provides suitable conditions for the formation of R-loops (Roy et al., 2010; Massé and Drolet, 1999). In return, R-loops can be used as a superhelix stress eliminator to return the negative superhelix domain to a more energetically favorable (lower) state, relaxing the remaining DNA structural domains in part or completely (Stolz et al., 2019). *In vitro* experiments on plasmids have shown that the relaxation efficiency of R-loops is tens of times higher than that of nucleosomes of the same length (Stolz et al., 2019).

#### 3.1.3 Gene expression regulation

**FIGURE 5:**
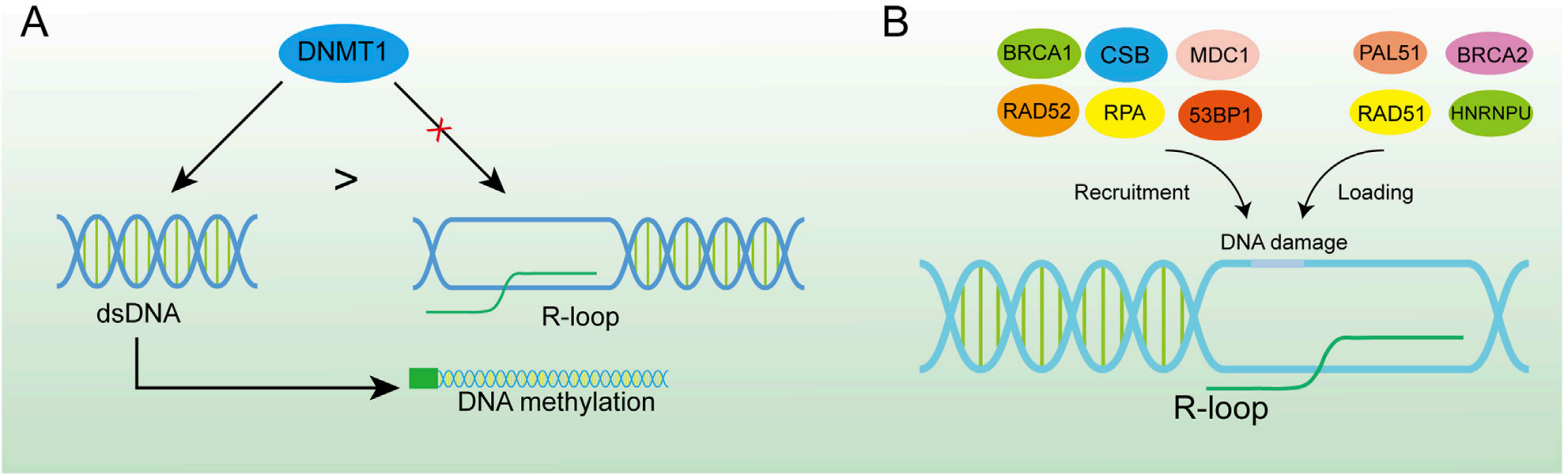
R-loop-induced DNA damage activates the DNA repair system. **(A)** DNMT1 has a higher binding force to dsDNA, and the formation of the R-loop in the promoter region can effectively inhibit DNA methylation. **(B)** R-loop is an important platform for recruiting BRCA1, CSB, MDC1, RAD52, RPA, 53BP1 and loading PAL51, BRCA2, RAD51, and HNRNPU at DNA damage sites which is of great significance for DNA damage repair.

Gene expression is the cornerstone of biological activities, and studies have shown that the R-loop regulates gene expression in a variety of ways (Ginno et al., 2012; Colak et al., 2014). In epigenetics, the R loop can influence the methylation process of many genes. Since many R-loops are often found on unmethylated CGI, their presence shields the promoter from the action of DNA methyltransferases, thus facilitating the transcription of downstream genes (Chedin, 2016). For instance, DNA methyltransferase 1 (DNMT1), as a vital enzyme in the methylation process, has a strong affinity for double-strand DNA (Mohan, 2022). However, the RNA-DNA hybrid in R-loops makes it difficult for DNMT1 to bind to the promoter region of the gene (Galbraith and Snuderl, 2022; Grunseich et al., 2018). The nascent RNA has a space-occupying effect, impedes DNA methylation and regulates gene expression (Figure 5A). The R loop can also reduce methylation levels of CGI by attracting ten-eleven translocation (TET) DNA demethylases, and thousands of R-loop-dependent TET1 binding sites are present on CGI in human embryonic stem cells (Arab et al., 2019). To identify the existence of R-loops in CGI promoters, the DRIP method combined with deep sequencing was used and verified the universality of R-loops (Ginno et al., 2013). This was further investigated by Christopher et al. who, by employing DRIP and methyl C-seq techniques, revealed that in human cells the R-loop promotes transcription of more than 1,200 genes by preventing DNA methylation-directed gene silencing (Grunseich et al., 2018). However, there is a contrary view that R-loop binding to the DNA promoter region may occupy the binding sites of transcription factors in DNA (Boque-Sastre et al., 2015). This would disrupt the transcription process and accelerate the disorder of downstream RNA synthesis and gene expression, which has a huge impact on the cell cycle (Mackay et al., 2020). Besides the abundant binding in promoter regions, R-loops also gather in the G-rich terminator (Zhao et al., 2016). This assists RNA polymerase Ⅱ to stop on the template DNA strand and is conducive to transcriptional termination, resulting in precise regulation of gene expression, which has been verified in several gene transposons (Zhao et al., 2016; Skourti-Stathaki et al., 2011). One study has demonstrated that the recruitment of the helicase SETX by the R-loop at the 3′end of the gene promotes efficient transcription termination (Giannini and Porrua, 2024). The interaction between the R-loop and the G-quadruplex (G4) as secondary structures of the cellular nucleic acid chain is of equal interest. The R-loop formed in transcription promotes mRNA production by facilitating the formation of G4 in the non-template strand of DNA on the one hand (Lee et al., 2020), and on the other hand has been shown to recruit CCCTC-binding factors (CTCF) in mouse embryonic stem cells, where CTCF proteins would regulate gene expression by binding to target DNA sequences (Wulfridge et al., 2023; Dehingia et al., 2022).

#### 3.1.4 DNA repair

Recently, R-loops critical role in DNA repair has been highlighted by increasing studies. Although excessive aggregation of R-loops in cells is a major cause of genomic instability, RNA-DNA hybrid was reported to promote homologous recombination (HR) or C-NHEJ between chromosomes, which is important for repairing DSBs in DNA (Refaat et al., 2023; Aguilera and Gómez-González, 2017). When R-loop levels are lower than normal, homologous recombination and NHEJ repair are less efficient (Ohle et al., 2016; Yasuhara et al., 2018). The occurrence of DSB is usually accompanied by the emergence of an R-loops structure. Given the loss of integrity of the DNA strand, the DNA repair requires NHEJ, and RNA polymerase II can be recruited through the MRE11-RAD50-NBS1 (MRN) complex for nascent RNA synthesis (Chang et al., 2019). This type of repair can cause DNA repair errors when the RNA strand in the R-loop can replace the damaged DNA strand and function as a template strand for DNA repair (Petermann et al., 2022). HR is the repair modality with the highest fidelity in DNA repair, especially in DSBs, and the R-loop acts mainly by facilitating HR (García-Muse and AguileraLoops, 2019; Xue and Greene, 2021). Moreover, the R-loop structure is also a robust recruitment platform for a variety of DNA damage repair factors (Figure 5B). The R-loop, as an essential molecule for efficient HR genesis, promotes the disappearance of DSBs through various pathways, although the general principle is similar: DSB induces the production of the R-loop, which in turn exerts its repair function by recruiting a series of DNA damage repair factors, such as RAD52 (Yasuhara et al., 2018), BRCA1 (D'Alessandro et al., 2018), RPA (Domingo-Prim et al., 2019), etc., and further enhances the loading of other repair factors, such as RAD51 (Wahba et al., 2013), BRCA2 (Teng et al., 2018), among which heterogeneous nuclear ribonucleoprotein U (HNRNPU) has been shown to interact with the R-loops to promote C-NHEJ-mediated DNA repair (Refaat et al., 2023). DNA repair factors and the corresponding hydrolases have been summarised (Table 1). The R-loops are subsequently hydrolysed by enzymes such as XPG (Yasuhara et al., 2018), SETX (Cohen et al., 2018a), RNase1 (Burger et al., 2019), RNase2 (Ohle et al., 2016) and dead-box helicase 1 (DDX1) (Li et al., 2016). Notably, some studies have found that Drosha enzymes are responsible for regulating the recruitment of repair factors to promote DNA repair. This suggests that Drosha enzymes may be related to the R-loops in some way, but further studies are needed (Camino et al., 2023; Liu et al., 2022).

**TABLE 1:** DNA repair factors associated with the R-loop.

	Factor	Recombination form	Ribonuclease
Recruitment	RAD52	HR	XPG
	BRCA1	HR	XPG
	CSB	HR	—
	53BP1	—	RNaseH1
	MDC1	—	RNaseH1
	RPA	HR	RNaseH1
Loading	BRCA2	HR	RNaseH2
	PALB2	—	—
	RAD51	HR	senataxin
	HNRNPU	C-NHEJ	—

RAD52: RAD52 Homolog, DNA, repair protein, BRCA1: BRCA1 DNA, repair associated; CSB, Cockayne syndrome group B, 53BP1: p53-binding protein 1, MDC1, Mediator Of DNA, Damage Checkpoint 1; RPA, Replication Protein A, BRCA2: BRCA2 DNA, repair associated; PALB2, Partner And Localizer Of BRCA2, RAD51: RAD51 Recombinase, Heterogeneous Nuclear Ribonucleoprotein U; HR, homologous recombination; C-NHEJ, Classical Non-Homologous End Joining; XPG, xeroderma pigmentosum, Complementation Group G.

### 3.2 Destructive role

#### 3.2.1 DNA injury

Targeting the DSBs pathway in tumor cells has become an important tool for cancer treatment in precision medicine (Gillyard and Davis, 2021). Numerous studies have shown that DSBs and R-loops are often causally related. R-loops can be involved in the induction of DSBs or can be induced by DSBs (Promonet et al., 2020; Tan et al., 2020b; Arnould et al., 2023). R-loops primarily cause DSBs by stalling replication forks: the stable R-loop persists in the genome and ssDNA, the template strand, is encapsulated by the R-loop structure, which temporarily loses its ability to act as a template. During subsequent genome replication, DNA polymerase II is unable to bind to the template strand to attach free deoxy-ribonucleoside triphosphate (dNTP), and the DNA strand cannot be formed, resulting in a discontinuous ssDNA containing gaps, loss of the double helix structure, and easy breakage of the DNA under stress at the gaps (Hamperl et al., 2017). In addition, the R-loop has been found to disrupt DNA structure through non-replication dependence in some types of cells (Sollier and Cimprich, 2015; Cristini et al., 2019). DNA topoisomerase I (TOP1) enzymes can unwind DNA superhelix during transcription by forming topoisomerase 1-DNA cleavage complexes (TOP1cc) (Chowdhuri and Das, 2021). For instance, in fibroblasts, scientists first discovered that removal of TOP1cc produces single-strand breaks (SSBs) intermediate and that DSBs are ultimately generated by R-loops, which are then subjected to cleavage by the enzymes XPF, XPG and flap structure-specific endonuclease 1(FEN1). Similarly, depletion of TOP1 enzyme and RNase H enzyme in yeast cells was observed to enhance the results of R-loop-mediated non-dependent replication to increase DSBs (Stuckey et al., 2015). However, scientists observed diametrically opposite results in mammary epithelial cells, indicating that the induction of DSBs by the TOP1 enzyme and the R-loop is a very complex process that requires further study (Hidmi et al., 2024).

#### 3.2.2 Genomic instability

The pernicious effects of R-loops on genome stability in bacteria and humans by interfering with DNA replication and causing DSBs or chromosomal translocations have been demonstrated in both eukaryotic and prokaryotic cells (Gan et al., 2011). A widely accepted theory is that the dissociation of ssDNA in the R-loop makes the base which was originally inside the double helix structure free outside and is directly exposed to various harmful substances, such as multiple nucleases and genotoxic compounds, which are sensitive factors for DNA damage (Paulsen et al., 2009). Another fact is that the occupation of the R-loop in the genome hinders the progress of the replication fork during replication, which may lead to replication fork breakage or stalling (Stirling et al., 2012), and for a prolonged period this prevents replication from restarting, which is consistent with the instability of the genome (Gan et al., 2011). Meanwhile, the imbalance in R-loops leads to increased sensitivity of DNA to damaging factors, making it easier to induce DNA damage (Sarkar et al., 2018). An example of this is that in B cells overexpressing RNase H, the “collapsed R-loop” is susceptible to the AID enzyme, which deaminates dC to produce dU, more likely to cause DSB and recombination (Yu and Lieber, 2019; Yu et al., 2003). Another mechanism of DNA damage caused by R-loops may be mediated by base-excision repair (BER), such as the recruitment of nucleotide excision repair factors RAD1/XPG and RAD2/XPF by R-loop aggregation (Su and Freudenreich, 2017; Safari et al., 2021). In the process of removing R-loops, both of them will produce base deletions and DSBs in the replication process, leading to genome damage and cell genome instability (Mackay et al., 2020). All these are the main sources and strong evidence of genomic instability caused by the R-loop.

## 4 R-loops are an important risk factor for many diseases

The role of pathological R-loops in human disease has become increasingly important with the shift in scientific understanding of R-loops and has been studied extensively in the last decade. Pathological R-loops are uncommon in cells but are predominantly found in cells with defects in regulatory function (Zhang S. et al., 2024). The persistence of R-loops destabilizes the cellular genome through two main mechanisms. First, the presence of R-loops increases the exposure of chemical reaction groups on the DNA strand, and the exposed non-transcriptional strand will be easily damaged by endogenous enzymes, resulting in base changes or abnormal chain structures (Zhang et al., 2023). The accumulation of R-loops can lead to the formation of transcription arrest-related complexes, blocking the progress of DNA replication bifurcation and normal transcription processes (Chan et al., 2014a). Obviously, it amplifies the defective cells' pathological state and exacerbates the disease phenotype (Zeman and Cimprich, 2014). In addition, pathological R-loops have been reported to contribute to disease development by activating innate immunity (Crossley et al., 2023). Homeostatic imbalances in cellular physiological functions result in a large number of pathological R-loops, which are one of the pathological features of many serious diseases, such as cancer, neurodegenerative diseases, severe hereditary disorders, aging-related disorders, myelodysplastic syndromes, childhood cancers, Ewing’s sarcoma, among others (Richard and Manley, 2017). To some extent, these diseases can be considered as R-loop-related diseases (Sanz et al., 2016; Chakraborty, 2020). The human diseases associated with the R-ring and the corresponding causative agents have been summarized. (Table 2).

**TABLE 2:** Human diseases associated with the R-loop.

Disease	R-loop factors	Assumed mechanism	References
ALS and FTD	C9ORF72	C9ORF72 repeat expansions induce aberrant R-loops and transcriptional interference, causing DNA damage	Farg et al. (2017)
ALS4	SETX	Dominant SETX mutations boost helicase expression, reduce R-loop levels, impair promoter methylation prevention, and lower gene expression	Grunseich et al. (2020)
AOA2		Recessive mutations in SETX reduce helicase activity, leading to elevated levels of pathological R-loops in AOA2 cells	Moreira et al. (2004)
ALS	TDP-43	Mutated TDP-43 can cause pathological R-loop accumulation through mislocalization or depletion via small interfering RNA (siRNA)	Giannini et al. (2020)
SMA	SMN1	The proteins encoded by these two genes interact to enhance pre-mRNA splicing efficiency. In contrast, defects in these genes impede pre-mRNA splicing, leading to R-loop formation and genomic damage	Ito et al. (2018b)
	ZPR1		Kannan et al. (2020)
MDS	SRSF2, U2AF1	Mutations affecting splicing factors result in the formation of R-loops, which induce genomic instability and diminish cell performance	Chen et al. (2018)
ICF	TERRA	TERRA-induced R-loop overaccumulation causes telomere depletion and genomic instability	Vaid et al. (2024)
Breast and ovarian cancers	BRCA1	Aberrant BRCA1 expression leads to insufficient R-loop processing and accumulation of co-transcribed DNA damage, thereby promoting tumor progression	Hatchi et al. (2015)
	BRCA2	BRCA2 aberrant expression interferes with the regulation of RNAPII, increases the formation of R-loop, and exacerbates genomic instability	Gondo et al. (2021)
		BRCA2 aberrant expression affects mRNA export, leading to an increase in R loops, further damaging the genome	Shivji et al. (2018b)
	Estrogen	It has a strong stimulatory effect on R-loop formation, thereby inducing DSB and genomic instability	Yager and Davidson (2006)
Mulitiple cancers	SRSF1	The downregulation of SRSF1 leads to the accumulation of R-ring and the cells enter the regulatory dysregulation state	Arif et al. (2023)
	APOBEC3B	Directly and significantly increases the number of R-loops and induces mutations as well as genomic instability	McCann et al. (2023)
	Bre1	Defective expression of Bre1 leads to aberrant R-loop production, which in turn initiates cellular replication stress and genomic destabilization	Chernikova et al. (2012)
	HRAS^V12^	Overexpression of HRAS^V12^ accelerates r-loop formation by up-regulating TBP.	Kotsantis et al. (2016)
Synovial sarcoma	SS18-SSX1	Fusion expression of oncoproteins leads to increased levels of R-loop, which in turn leads to replication stress	Jones et al. (2017)
Ewing’s sarcoma	EWS-FLI, BRCA1	The R-loop originates from the transcriptional promotion of oncoproteins, leading to inhibition of BRCA1 function and consequent DNA damage	Gorthi et al. (2018)
Kaposi’s sarcoma	hTREX	ORF57 tightly binds to the export complex (hTREX) protein and inactivates it, ultimately leading to the accumulation of pathogenic R-loops	Jackson et al. (2014)
AML and MDS	DDX41	Mutations leads to accumulation of R-loop, replication stress, DSB, and remodeling of inflammatory signaling pathways	Mosler et al. (2021)

ALS, amyotrophic lateral sclerosis; FTD, frontotemporal dementia; AOA2, Ataxia with oculomotor apraxia 2; SMA, spinal muscular atrophy; MDS, myelodysplastic syndromes; ICF, immunodeficiency; centromere instability, and facial anomalies syndrome; AML, acute myeloid leukemia.

### 4.1 Neurodegenerative disorders

The abnormal management of R-loop metabolism is a prevalent underlying mechanism that mediates the degeneration of neurons in a range of neurodegenerative conditions, leading to disease manifestation (Perego et al., 2019; Richard and Manley, 2014; Ma et al., 2018; Cohen et al., 2018b; Groh et al., 2017; Richard et al., 2021).

#### 4.1.1 Amyotrophic lateral sclerosis (ALS)

ALS is the most common neurodegenerative disease, characterized by the loss of motor neurons in the brain and spinal cord, resulting in muscle weakness or atrophy (Al-Chalabi and Hardiman, 2013). Several genes are involved in ALS pathogenesis, but R-loop involvement in ALS is not negligible (Cappella et al., 2019). The hexanucleotide GGGGCC (G4C2) amplification in the C9ORF72(C9) gene is the most prevalent genetic cause of ALS, and previous studies have shown that the C9 repeat amplification induces the DNA damage response (DDR) in ALS (Farg et al., 2017). The number of genomic CGI is significantly higher in some ALS patients than in normal subjects, and these duplicated CGI transcriptionally generate the R-loops (Perego et al., 2019; Reddy et al., 2014). Although the role of CGI in the formation of pathological R-loops is unclear, persistent R-loops certainly induce DNA damage in neurons affecting the stability of repetitive nucleotide sequences and even entire genomes (Reddy et al., 2014; Konopka and Atkin, 2018). This is supported by the finding of Manal et al. that in ALS, the expansion of the C9 repeat activates the DDR in neurons (Farg et al., 2017). Ultimately, this R-loop-dependent DNA damage leads to the degeneration and even the death of nerve cells in the brain.

#### 4.1.2 ALS4 and ataxia with oculomotor apraxia type 2 (AOA2)

ALS4 is a subgroup of ALS, which is characterized by the fact that it affects adolescents and progresses slowly (Grunseich et al., 2020). AOA2 is an autosomal recessive disorder in which patients often present with polyneuropathy and microcephaly (Anheim et al., 2009). The two diseases are caused by different mutations in the SETX gene (Moreira et al., 2004). SETX is a highly conserved RNA/DNA helicase that can function by mediating R-loop resolution (Mischo et al., 2011). When a dominant mutation of SETX occurs, the expression of helicase increases accompanied by an increase in the degradation of R-loops, resulting in ALS4 (172). The detailed mechanism is that the mutation of SETX decreases its expression, the abundance of R-loops in the promoter region of the BMP and activin membrane-bound inhibitor (BAMBI) gene decreases, the effect of preventing promoter methylation weakens and gene expression decreases (as mentioned above), while downregulation of BAMBI protein specifically activates the TGF-β pathway and finally contributes to the occurrence of ALS4 (Grunseich et al., 2018). TDP-43 is an RNA-binding protein whose depletion sensitizes neurons to DSBs (Mitra et al., 2019). According to clinical statistics, dominant mutations in the TDP-43 gene have a 4% and 1% incidence of familial and sporadic ALS, respectively (Sreedharan et al., 2008). In ALS4 patients, mutant TDP-43 was found to cause accumulation of R-loops through mislocalization or small interfering RNA (siRNA) depletion, with subsequent pathological R-loops exacerbating the disease phenotype by producing DSBs or promoting TRCs (Giannini et al., 2020). Notably, pathological changes in TDP-43 localization were caused by acquired mutations in SETX, indicating that SETX may contribute to the ALS disease pathway by indirectly modulating R-loop levels (Bennett et al., 2018). In contrast, when there is a recessive mutation in the SETX gene, helicase levels are reduced and a large number of body-damaging R-loops are produced in the cell, ultimately leading to AOA2 (Becherel et al., 2015). In addition, compared to ALS4, AOA2 cells also block SETX from interacting with the exosome subunit Rrp45, inhibiting exosomes from functioning (Richard and Manley, 2014; Richard et al., 2013). Normally, exosomes are responsible for breaking down the RNA produced by SETX de-localization (Houseley et al., 2006); therefore, the remaining RNA in AOA2 cells would still have the potential to exacerbate the disease phenotype.

#### 4.1.3 Spinal muscular atrophy (SMA)

**FIGURE 6:**
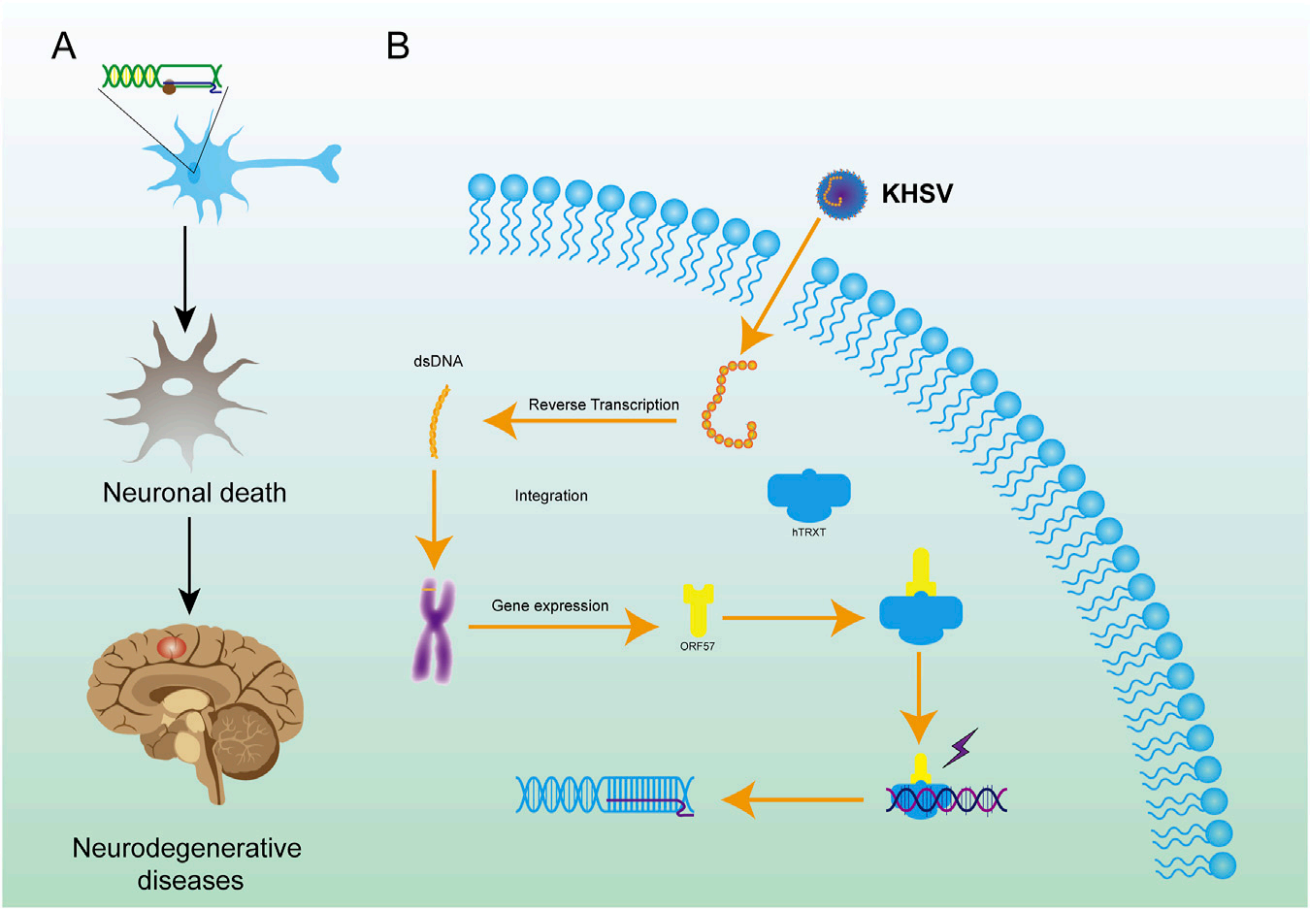
Mechanisms of R-loop formation by KHSV in cells and the role of R-loop in neurodegenerative diseases. **(A)** KHSV infection can integrate the genome into host cells, leading to the formation of R-loop. **(B)** The R-loop structure in neurons causes irreversible damage to neurons, leads to the death of neurons, and finally leads to neurodegenerations.

SMA is a recessive genetic disorder caused by mutations in the motor neuron 1 (SMN1) gene on chromosome 5q13 and is characterized by muscle atrophy caused by degeneration of motor neurons in the spinal cord (Wirth, 2000). Mutations in the ZPR1 zinc finger (ZPR1) or SMN1 genes in the cellular genome were screened in SMA patient cells by deep sequencing. The proteins encoded by these two genes interact intracellularly to increase the efficiency of pre-mRNA splicing (Ito et al., 2018a). This can induce the differentiation of neurons and promote the growth of axons. Both gene defects contribute to the obstacle of pre-mRNA progression, produce R-loops, and damage the cell genome (Figure 6A) (Kannan et al., 2020; Kannan et al., 2022).

### 4.2 Cancer

Although there is no direct evidence of the relationship between R-loops and tumor cells, the elevated R-loops content observed in tumor cells suggests that R-loops may play a role in the progress of tumors. The R-loop is one of the main sources of replication stress, and replication fork collapse under stress, especially in TRC, is a phenomenon triggered by oncogenic expression (Helmrich et al., 2011; Kotsantis et al., 2016). Many carcinogenic events have been proven to promote the formation of R-loop in cells. The hormone estrogen (E2) promotes the transcription of E2-related genes and evidence shows there is a high correlation between elevated levels of E2 and breast cancer (BRCA) (Yager and Davidson, 2006). E2 also has a strong stimulation on the R-loops formation which induces DSBs and genomic instability (Stork et al., 2016). HRAS^V12^ as an oncogene promotes the synthesis of intracellular *de novo* RNA and stimulates the proliferation of cells (Di Micco et al., 2006). Furthermore, overexpression of HRAS^V12^ accelerates the formation of R-loops by upregulating TATA-box binding protein (TBP) which is a source of stagnation of replication fork and DNA injury (Kotsantis et al., 2016). E3 ligase Bre1 plays a vital role in biological processes such as DNA replication, transcription elongation, and genome repair through mono-ubiquitination of histone H2B (Liu et al., 2021; Wu et al., 2017). Depletion of Bre1 homologs causes genomic instability due to R-loop accumulation and the potential development of testicular seminoma (Chernikova et al., 2012). Moreover, many other oncoproteins participate in the process of cell malignant transformation through various pathways, like EWS-FLI1 (Gorthi et al., 2018), SS18–SSX1 (Jones et al., 2017), etc. All the evidence mentioned above indicates that there is a molecular link between R-loop formation, genomic instability, and tumorigenesis. Many factors influence the abundance of R-loops in cells, as we have concluded previously, and these factors may then directly or indirectly influence tumorigenesis through the R-loop-associated DNA damage pathway and others. Apart from R-loop-induced genomic instability, oncogene-induced replication stress is another significant mechanism of genomic instability caused by DDR (Graziano and Gonzalo, 2017; Petropoulos et al., 2019; Chan et al., 2014b).

Mutations in transcription factors can mediate the malignant transformation of cells through the R-loop pathway and promote cancer development (Zatreanu et al., 2019; Bhattacharjee et al., 2024). In the case of transcriptional regulation, deletion of the gene serine and arginine-rich splicing factor 1(SRSF1) leads to a reduction in the function of its regulatory genes, which directly damages cells (Arif et al., 2023). In particular, the downregulation of SRSF1 contributes to the formation of R-loops during transcription (as described above), and the accumulation of R-loops in the cell leads to DSBs, DNA mutations, RNA polymerase sensitivity, replication fork stalling, and chromosome breaks, which in turn affect the stability of the genome and the cell enters a state of regulatory dysregulation leading to cancer oncogenesis (Olazabal-Herrero et al., 2024). Notably, during transcription, apolipoprotein b mRNA editing enzyme catalytic subunit 3B (APOBEC3B) can directly induce a striking number of R-loops to produce gene mutations, and such mutated R-loops are more inclined to lead to genomic instability and to drive tumorigenesis (McCann et al., 2023).

The accumulation of R-loops can also activate the expression of telomerase, prolonging telomere length and enabling cells to obtain unlimited proliferation ability and tumor transformation (Shiromoto et al., 2021). ROS-mediate telomeric DNA damage can lead to the accumulation of R-loops, which further leads to genomic instability and cancer (Tan et al., 2020b; Tan and Lan, 2020). In the absence of telomerase and homology-directed repair (HDR), the reduced ability of telomeres to self-replicate leads to telomere shortening and accelerated senescence, which is a major pathogenic factor in neurodegenerative diseases (Wang J. et al., 2020; Rahman et al., 2019). In the presence of HDR, the accumulation of the RNA-DNA hybrid double strand can promote telomere transcription and telomere recombination (Balk et al., 2014). RAD51 promotes the formation of telomeric R-loops by accelerating the binding of endogenous TERRA and telomeres, which may result in telomere fragility (Feretzaki et al., 2020; Ghisays et al., 2021). A recent study has shown that low m6A methylation results in telomere damage due to the inability of the R-loop to direct the TERRA lncRNA to target telomeres by RNA modification (Vaid et al., 2024).

#### 4.2.1 Kaposi’s sarcoma

Viral infection can also cause malignant transformation of cells, which directly affects the integration of the viral genome into cell chromosomes and results in unregulated cell growth (D'Souza et al., 2020). Secondly, viral infection can result in the accumulation of R-loops in cells by inhibiting the expression of related proteins, leading to the malignant transformation of cells (Templeton and Laimins, 2023; Kumari et al., 2023). For example, Kaposi’s sarcoma-associated herpes virus (KSHV) is carcinogenic (Sandhu and Damania, 2022). The virus expresses the ORF57 protein, which is a regulatory factor of viral mRNA processing. ORF57 can tightly bind the export complex (hTREX) protein and inactivate it by masking the binding sequence, preventing it from participating in mRNA processing. This causes R-loops to accumulate in cells, leading to cancer (Figure 6B) (Jackson et al., 2014). In addition to KSHV, other viruses such as human papillomavirus (HPV), and Burkitt’s lymphoma also damage the genome directly or indirectly after infection (Templeton and Laimins, 2023; Kumari et al., 2023).

#### 4.2.2 Blood disease

Hematopoietic stem and progenitor cells (HSPCs) have the capacity for long-term self-renewal and the potential to differentiate into various types of mature blood cells, the homeostasis of which is of great significance to the hematological system. The abundance of the R-loop has been found to affect the stability of HSPCs and has been implicated in acute myeloid leukemia (AML), myelodysplastic syndromes (MDS), adult T-cell leukemia and B-cell lymphoma (Frame and North, 2021; Weinreb et al., 2021; Lee et al., 2023; Chen et al., 2018; He et al., 2021). Among them, activation of DDX41-inflammation pathways is one of the important approaches to R-loop-induced disease progression. Dead-box helicase 41 (DDX41), a helicase, not only unwinds DNA-RNA hydrids but also activates the cGAMP synthase (cGAS)-Stimulator of Interferon Genes (STING) pathway in blood disorders (Winstone et al., 2024; Smith et al., 2023). Mutant DDX41 in myeloid cells results in the accumulation of pathological R-loops causing genomic instability and aberrant activation of the inflammatory response pathway (cGAS-STING), ultimately leading to AML or MDS (Mosler et al., 2021). In addition, aberrant expression of lncRNAs like HOTTIP (Luo et al., 2022), epigenetic like m6A methylation (Hwang et al., 2024), and transcriptional regulators like SRSF2 and U2 small nuclear RNA auxiliary factor 1 (U2AF1) can lead to abnormally elevated levels of R-loops and ultimately to blood disorders (Pellagatti and Boultwood, 2020).

## 5 Challenges and prospects in applying R-loops to the clinic

In view of the extensive roles of R-loops in cells and their impact on genome stability, potential clinical applications of the latest R-loops content detection methods are explored for their possible roles in predicting the onset of diseases, including some genome-related diseases. Clinical predictive markers are used as the basis for the diagnosis of certain diseases in molecular biological examinations (Antoranz et al., 2017). However, current laboratory methods for detecting R-loops are imperfect and do not permit accurate and quantitative detection of R-loops as a clinical predictive molecule. The work of Zhang et al. supports that the R-loop score can be used to predict the prognosis of patients with pan-cancer, but there is limited evidence that the R-loop itself causes cancer progression. More extensive analyses and validation are needed to determine whether the R-loop can be used as a biomarker of disease (Zhang S. et al., 2024). Therefore, it is necessary to explore other methods that can determine R-loop content accurately, simply, and rapidly before this potential marker can be applied in clinical practice. A broad range of methods have been developed to detect R-loop abundance, including traditional DRIP-seq (Ginno et al., 2012), as well as DRIPc-seq (Sanz et al., 2016) and ULI-ssDRIP-seq (Xu et al., 2024) techniques. Interestingly, Magdalena et al. used recombinant catalytically inactivated green fluorescent protein-RNase H1 (GFP-dRH) to visualize the DNA-RNA hybrid (Crossley et al., 2023). As the interaction of SETX with the R-loop is associated with a variety of diseases, we propose to visualize the R-loop using recombinant inactivated SETX proteins with fluorescent tags. The feasibility of this approach needs to be further validated, but it is theoretically more suitable for studying the pathogenesis of the R-loop in disease.

Simultaneously, disease prevention and treatment might be achieved by adjusting certain sites on the R-loop generation pathway. By regulating the activity of related enzymes, the negative impact of R-loops on the cell genome could potentially be eliminated to achieve the purpose of clinical treatment. This may be an effective treatment direction for some genomic abnormalities.

Hypermethylation on the C9 promoter has been observed in ALS-diseased mice and people (Esanov et al., 2017; Xi et al., 2013). Representatives are heterochromatin with H3K9me3 or H3K26me3, which act to repress excessive C9 transcription in cells (Wang et al., 2018; Belzil et al., 2013). It was previously suggested in the literature that the R-loop might achieve a delay in ALS progression by modulating chromatin modifications (Wang et al., 2015), but it has been definitively shown that the R-loop is not significantly associated with hypermethylation (Esanov et al., 2017). Paradoxically, most studies point to DNA-RNA hybrids avoiding the action of DNA methylases (as described above), so further studies are needed to determine why there are high levels of R-loops in hypermethylated C9 genes.

R-loops are involved in the initiation and development of related diseases through a variety of mechanisms including genetic disorders, neurodegeneration, and cancer. While R-loops are instrumental in the occurrence of such diseases, they are also an important target for treatment (Tan and Lan, 2020). Taking tumors as an example, excessive accumulation of R-loops promotes genomic instability and may result in the malignant transformation of cells. Through the exploration of the role of R-loops in the pathogenesis of cancer, we can treat the tumor cells by altering the level of R-loops, changing the relative pathway of tumor cells, and even inducing tumor cell apoptosis directly (Boros-Olah et al., 2019). Loss of bromodomain containing 4 (BRD4) can inhibit many cellular events to reduce the accumulation of R-loops, and has proved useful in the treatment of cancers (Lam et al., 2020b). By parity of reasoning, it is concluded that by targeting many genes and factors, tumor cell death can be induced clinically through modulating the abundance of the R-loop, which would be a good way to treat tumors associated with the R-loop.

Recent studies have shown that many diseases are directly or indirectly related to R-loops (Khan and Danckwardt, 2022). As a widespread gene regulation mechanism in cells, many diseases could potentially be treated or prevented based on the regulation mechanism of R-loops. The aim of such disease treatments would be to maintain the homeostasis of R-loops in cells. In view of the dynamic process of R-loop contents in the body, nascent R-loops are constantly produced, accompanied by the continuous degradation of R-loops, making it in a dynamic equilibrium state. By accelerating the formation and degradation of R-loops, the content of these structures can be kept in a transient and stable state, which is conducive to their role in the body, avoiding rapid accumulation and adverse effects on the collective. Regulation of R-loops may therefore be a novel therapy to control disease and can be achieved by promoting the synthesis or degradation of R-loops with the goal of achieving gene therapy.

## 6 Conclusion

As normal structures in cells, R-loops participate in the regulation of many basic cellular activities. However, deepening research on R-loops has uncovered more and more novel functions and mechanisms of these hybrid nucleic acid structures. The content of R-loops is closely associated with many severe diseases, sometimes R-loops themselves are even the direct causes of diseases. To fully understand these diseases and identify possible treatment methods, new functions of R-loops must be constantly explored. Increasing numbers of genes are reported to be closely related to the abundance of R-loops. Furthermore, the pathogenesis of many diseases can be explained by R-loops. Such diseases can potentially be treated or even prevented from the R-loops level based on the huge potential of R-loops.

Many factors affect the level of R-loops in cells. The factors above-mentioned are only a small part of those that have been verified, and many other mechanisms have not yet been explored. Further dissection of the mechanism of R-loop formation promises to yield many approaches to adjust the abundance of R-loops to normal levels and prevent the negative effects of R-loops in cells or to increase R-loop abundance in malignant cells to mediate cell death. This may have major implications in the prevention and treatment of many diseases.

R-loops are one predominant regulator of many genetic instability diseases and have gradually received more attention as a target for clinical prediction and treatment. The detailed mechanism of genomic instability in cells has not yet been fully elucidated, and requires further exploration at the molecular level. In summary, many aspects of R-loops have not yet been fully revealed and need further research. For example, a more sensitive and convenient method is needed for R-loops detection, a comprehensive understanding of R-loops regulatory network and the effect of R-loops regulation in many diseases. We can further achieve the goal that predicting certain diseases and even curing some diseases by regulating R-loops, as well as providing more diverse options for clinical treatments.

## Glossary

RE: restriction endonuclease
dsDNA: double-stranded DNA
CSR: class switch recombination
HR: homologous recombination
NHEJ: non-homologous end joining
RRMs: RNA recognition motifs
DSBs: double-strand breaks
DRIP: RNA-DNA immunoprecipitation
TERRA: telomeric repeat-containing RNA
CGI: CpG island
G4: G-quadruplex
ROS: Reactive oxygen species
SRSF1: Serine/arginine-rich splicing factor 1
RRMs: RNA recognition motifs
PNPT1: polyribonucleotide nucleotidyltransferase 1
mtDNA: mitochondrial DNA
RER: ribonucleotide excision repair
SETX: senataxin
AQR: Aquarius
DDX: DEAD-box helicase
RTC: replication-transcription collision
PTM: post-transcriptional modification
RNAPII: RNA polymerase II
PPP: promoter-proximal pausing
AID: activation-induced deaminase
NHEJ: non-homologous end-joining
DNMT1: DNA methyltransferase 1
TET: ten-eleven translocation
CTCF: CCCTC-binding factors
HR: homologous recombination
MRN: MRE11-RAD50-NBS1
HNRNPU: heterogeneous nuclear ribonucleoprotein U
TOP1: DNA topoisomerase I
SSBs: single-strand breaks
FEN1: flap structure-specific endonuclease 1
BER: base-excision repair
ALS: amyotrophic lateral sclerosis
FXS: fragile X syndrome
RPA: replication protein A
DDR: DNA damage response
siRNA: small interfering RNA
KSHV: Kaposi’s sarcoma-associated herpes virus
HPV: human papillomavirus
BER: base-excision repair
AOA2: apraxia type 2
CGI: CpG island
HDR: homology-directed repair
