# Supplementary figures and images for: Mechanisms underlining R-loop biology and implications for human disease
Source: Front Cell Dev Biol. 2025 Feb 21;13:1537731. doi: 10.3389/fcell.2025.1537731 (PMC11885306; doi:10.3389/fcell.2025.1537731)

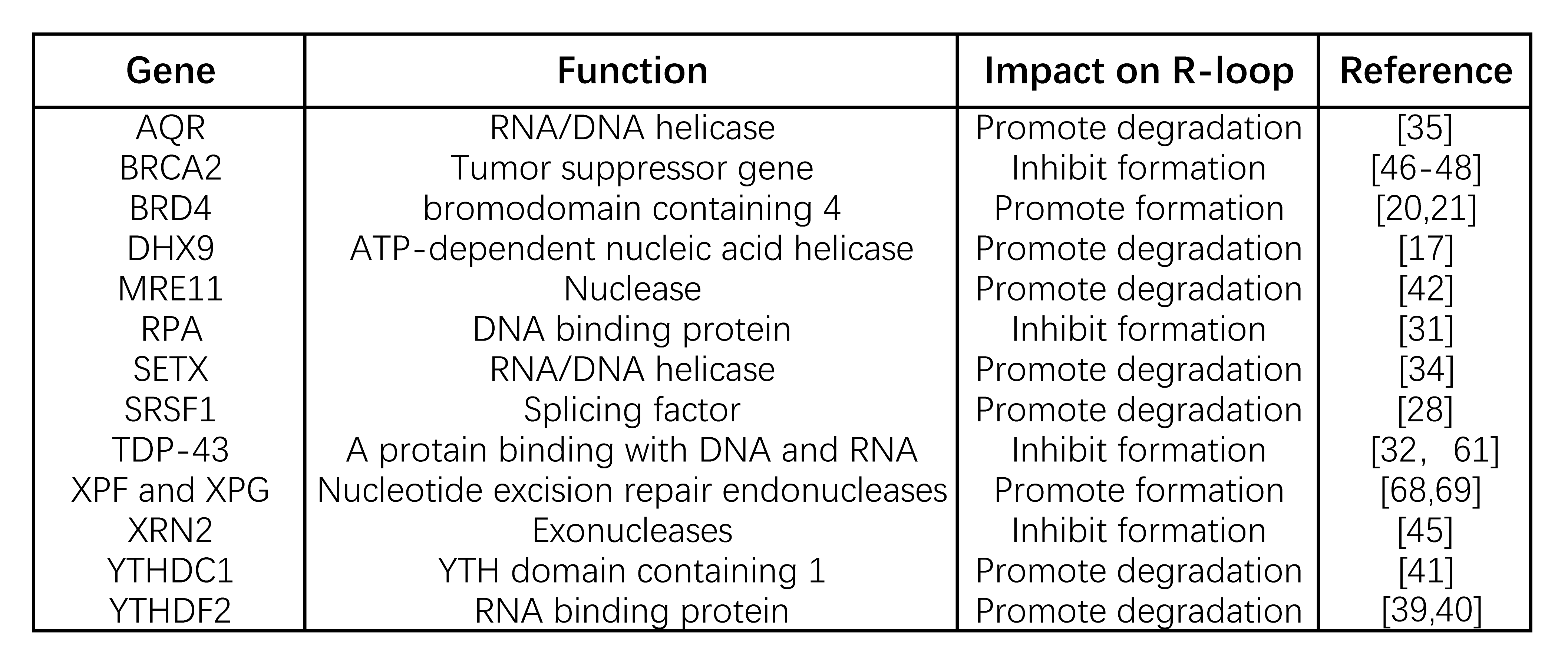

Supplement: Supplementary file 1 [file Image1.tif]
